# A speed–fidelity trade-off determines the mutation rate and virulence of an RNA virus

**DOI:** 10.1371/journal.pbio.2006459

**Published:** 2018-06-28

**Authors:** William J. Fitzsimmons, Robert J. Woods, John T. McCrone, Andrew Woodman, Jamie J. Arnold, Madhumita Yennawar, Richard Evans, Craig E. Cameron, Adam S. Lauring

**Affiliations:** 1 Division of Infectious Diseases, Department of Internal Medicine, University of Michigan, Ann Arbor, Michigan, United States of America; 2 Department of Microbiology and Immunology, University of Michigan, Ann Arbor, Michigan, United States of America; 3 Department of Biochemistry and Molecular Biology, Pennsylvania State University, University Park, Pennsylvania, United States of America; 4 Department of Epidemiology, University of Michigan, Ann Arbor, Michigan United States of America; ETH Zurich, Switzerland

## Abstract

Mutation rates can evolve through genetic drift, indirect selection due to genetic hitchhiking, or direct selection on the physicochemical cost of high fidelity. However, for many systems, it has been difficult to disentangle the relative impact of these forces empirically. In RNA viruses, an observed correlation between mutation rate and virulence has led many to argue that their extremely high mutation rates are advantageous because they may allow for increased adaptability. This argument has profound implications because it suggests that pathogenesis in many viral infections depends on rare or de novo mutations. Here, we present data for an alternative model whereby RNA viruses evolve high mutation rates as a byproduct of selection for increased replicative speed. We find that a poliovirus antimutator, 3D^G64S^, has a significant replication defect and that wild-type (WT) and 3D^G64S^ populations have similar adaptability in 2 distinct cellular environments. Experimental evolution of 3D^G64S^ under selection for replicative speed led to reversion and compensation of the fidelity phenotype. Mice infected with 3D^G64S^ exhibited delayed morbidity at doses well above the lethal level, consistent with attenuation by slower growth as opposed to reduced mutational supply. Furthermore, compensation of the 3D^G64S^ growth defect restored virulence, while compensation of the fidelity phenotype did not. Our data are consistent with the kinetic proofreading model for biosynthetic reactions and suggest that speed is more important than accuracy. In contrast with what has been suggested for many RNA viruses, we find that within-host spread is associated with viral replicative speed and not standing genetic diversity.

## Author summary

Why organisms have different mutation rates is a longstanding question in evolutionary biology. The polymerases of RNA viruses generally lack proofreading activity and exhibit extremely high mutation rates. Because most mutations are deleterious and mutation rates are typically tuned by natural selection, we asked why RNA viruses haven’t evolved a polymerase with a lower mutation rate. We used experimental evolution and a murine infection model to show that RNA virus mutation rates may actually be too high and are not necessarily adaptive. Rather, our data indicate that viral mutation rates have evolved to be higher as a result of selection for viruses with faster replication kinetics. We suggest that viruses have high mutation rates, not because they facilitate adaptation but because it is hard to be both fast and accurate and these viruses have prioritized speed over fidelity.

## Introduction

Mutation is the ultimate source of genetic variation, and mutation rates can have a significant impact on evolutionary rate [1–3]. The intraspecific variability in mutation rate in many viruses and bacteria indicates that mutation rates have been optimized by natural selection [4–13]. Given that most mutations are deleterious, the burden of excess mutational load will select against strains with abnormally high mutation rates [14–17]. This principle led Sturtevant to ask, “Why does the mutation rate not evolve to zero?” [18,19].

A large body of theoretical and experimental work suggests that the selective pressure for higher mutation rates is due to either the physicochemical cost of maintaining a lower one or a selective advantage from an increased supply of beneficial mutations [20–23]. Many have argued for the adaptive benefit of high mutation rates in pathogenic microbes, which often exist in dynamic environments and are subject to host immune pressure [7,24,25]. However, direct selection of a variant with a higher mutation rate will only occur if it has been advantageous in the past, and in many cases, it has been difficult to separate the causes of a higher mutation rate from its consequences [19,26].

RNA viruses are ideal systems for studying the selective forces that act on mutation rates. While interspecies mutation rates range from 10^−4^ to 10^−6^ errors per nucleotide copied [4], studies of antimutators and hypermutators suggests that fidelity can only vary by several-fold within a species [27]. The severe burden of mutational load exerts a strong downward pressure on mutation rates, and hypermutator strains are attenuated in vivo [9,28–31]. Given the short generation times and remarkable fecundity of many RNA viruses, a small kinetic cost to higher fidelity should result in strong selection against antimutators [32,33]. However, the observed attenuation of antimutator RNA viruses in vivo has led many to argue for the adaptive benefit of high mutation rates, as genetic diversity provides a rich substrate for a virus’s evolution in the face of varying intrahost environments [7,10,34–38]. This concept is central to viral quasispecies theory, which generally proposes a link between genetic diversity and viral fitness [24,25].

Here, we define the selective forces that shape viral mutation rates by studying an antimutator variant. The 3D^G64S^ mutant of poliovirus was selected after serial passage in ribavirin, an RNA virus mutagen. The RNA-dependent RNA polymerase (RdRp, 3D) of this variant contains a single glycine to serine substitution [5–7]. The basal mutation rate of 3D^G64S^ is reported to be approximately 20% to 30% that of wild-type (WT) virus. While the 3D^G64S^ mutant is attenuated in poliovirus receptor (PVR) transgenic mice, the relative importance of replicative speed and fidelity to this phenotype is not clear [7,36]. Biochemical assays of 3D^G64S^ suggest a physicochemical cost of high fidelity, but as in other systems, its contribution to overall fitness remains unquantified [6,19,39].

## Results

**Fig 1:**
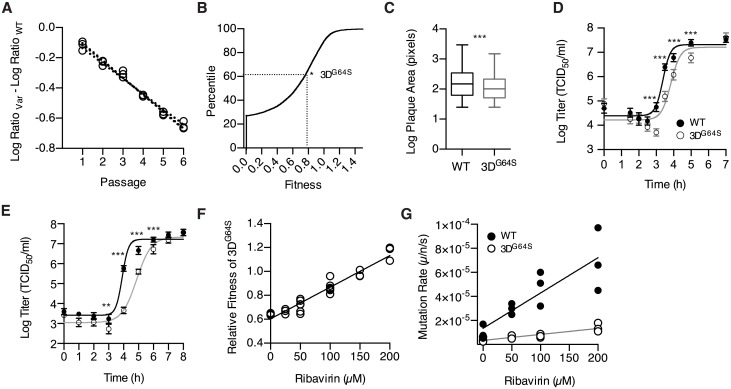
A speed–fidelity trade-off in the poliovirus RdRp. (A) Relative fitness of 3D^G64S^ as measured by direct competition. The amount of each virus at each passage was compared to the input and expressed as the difference in the log_10_ ratio in RNA genomes for 3D^G64S^ (open circles) relative to WT over time. The slope of the dotted lines are the relative fitness values, 0.78 ± 0.01, *n* = 3 replicates. (B) Cumulative distribution function of fitness values for SNVs of poliovirus as determined in [16]. “*” indicates the relative fitness (0.78) and percentile (64th) of 3D^G64S^. (C) Plaque size of clones from WT (*n* = 272; black) and 3D^G64S^ (*n* = 220; grey) virus populations. Box plots show median, 25% and 75% quartiles, and 1.5× interquartile range. ***p ≤ 0.005; *t* test with Welch’s correction. (D) Single-cycle growth curve for WT (filled circles, black line) and 3D^G64S^ (open circles, grey line) in HeLa. Data are mean ± standard deviation (*n* = 5 replicates). ***p < 0.005; unpaired *t* test comparing WT and 3D^G64S^ separately for each time point. (E) Single-cycle growth curve for WT (filled circles, black line) and 3D^G64S^ (open circles, grey line) in 3T3 cell line derived from MEFs of PVR transgenic mice. Data are mean ± standard deviation (*n* = 5 replicates). **p < 0.01; ***p < 0.005; unpaired *t* test comparing WT and 3D^G64S^ separately for each time point. (F) Relative fitness of 3D^G64S^ (open circles) as measured by competition assay (see panel A) in the presence of varying concentrations of ribavirin. Note that the baseline relative fitness of 3D^G64S^ (y-intercept) is lower than the fitness reported in panel A because the assays were performed under different experimental conditions (see Methods). (G) Mutation rate in mutations per nucleotide per strand copied for WT (filled circles) and 3D^G64S^ (open circles) in the presence of varying concentrations of ribavirin, as determined by Luria Delbruck fluctuation test. All plotted data can be found in S1 Data. HeLa, human epithelial cells; MEF, mouse embryonic fibroblast; PVR, poliovirus receptor; RdRp, RNA-dependent RNA polymerase; SNV, single-nucleotide variant; WT, wild-type.

We measured the relative fitness of 3D^G64S^ by direct competition over serial passage by quantitative reverse transcription polymerase chain reaction (qRT-PCR) (Fig 1A). Here, the fitness of 3D^G64S^ is 0.78 ± 0.01 (*n* = 3 replicates) relative to WT. This is a moderate fitness defect, falling in the 64th percentile in a dataset of 8,970 fitness values obtained for point mutants of poliovirus under similar conditions [16] (e.g., human epithelial cells [HeLa] multiplicity of infection [MOI] 0.1, 8-hour infection cycle, and 6 passages; Fig 1B). We also measured the relative growth properties of WT and 3D^G64S^ using a plaque-size assay, which measures the growth, burst size, and spread of individual viruses in the absence of competition [40–42]. The distribution of clonal plaque sizes was significantly different (*p* < 0.005; unpaired *t* test with Welch correction; *n* = 272 WT and *n* = 220 3D^G64S^ plaques) and consistent with a moderate fitness defect in 3D^G64S^ (Fig 1C). In contrast with prior work, we were able to detect a significant replication defect for 3D^G64S^ by one-step growth curve, but only with rigorous synchronization, more frequent time points, and larger numbers of replicates (Fig 1D). This replication defect was not specific to HeLa because we observed a similar lag for 3D^G64S^ in a 3T3 cell line that we derived from mouse embryonic fibroblasts (MEFs) from PVR mice (Fig 1E). These data demonstrate that the fitness defect of 3D^G64S^ is largely attributable to its slower replicative kinetics and is consistent with biochemical assays on purified RdRp [6,39].

The reduced mutation rate and replicative fitness of 3D^G64S^ suggest a trade-off between speed and fidelity in RNA virus replication. Here, the fitness gain from increased replicative speed is offset by a reduction in fitness due to increased mutational load. We derived a quantitative model of this trade-off (see S1 Text Model 1) by measuring the replicative fitness (Fig 1F) and mutation rate (Fig 1G, S1 Table) of WT and 3D^G64S^ under exposure to an exogenous mutagen, ribavirin [43]. WT and 3D^G64S^ had equal fitness at approximately 150 μM ribavirin. Based on these data, our model indicates that WT incurs a fitness cost of 0.137 from mutational load alone. Therefore, any fitness benefit of the high baseline mutation rates in WT would presumably need to offset this cost. In 3D^G64S^, the cost of mutational load is reduced to 0.037.

**Fig 2:**
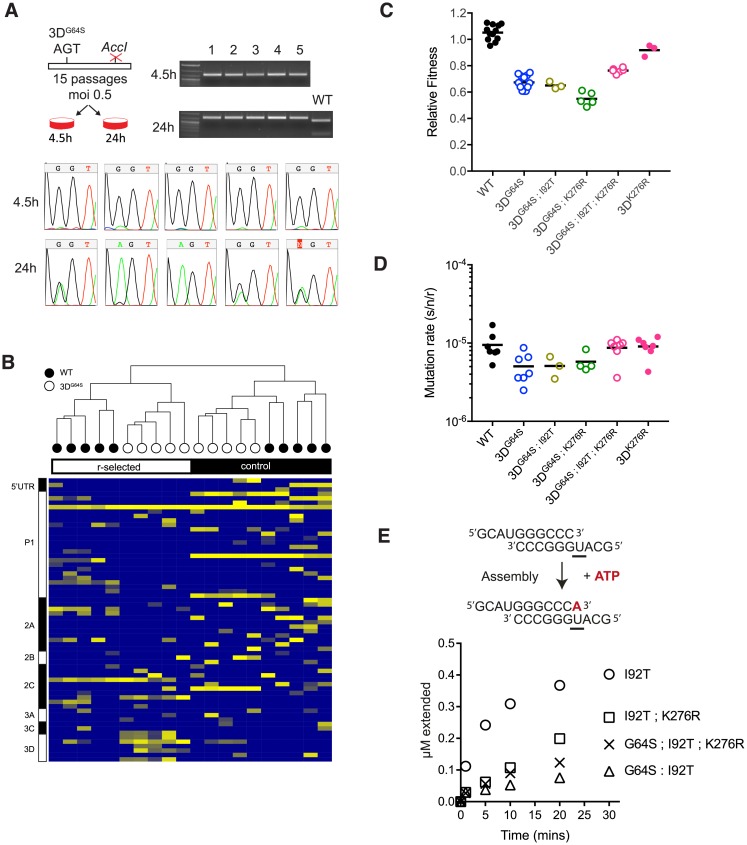
R-selection leads to increased mutation rates. (A) A point mutant of 3D^G64S^ (GGT^gly^ to AGT^ser^) was introduced into a poliovirus genome that is marked with a nearby point mutation that ablates an *AccI* restriction site. Viruses were serially passaged every 4.5 hours (r-selected) or every 24 hours (control) for 15 passages. Chromatograms show the codon for position 64 (either GGT^gly^ or AGT^ser^). Gel image of *AccI* restriction digest of all passage 15 populations showing that the reversion occurred in the parental backbone and was not due to contamination with WT virus, which retains the *AccI* site. (B) WT and a “locked in” version of 3D^G64S^ (GGT^gly^ to UCA^ser^) were subjected to r-selection (3.5–4 hours and 4–4.5 hours, respectively) or control (24-hour) passages for 50 passages as described in the text. Heatmap shows all mutations identified at >0.025 frequency in ≥2 out of the 20 total lineages, colored by log frequency. Diagram at left shows regions of the poliovirus genome. (C) Fitness of indicated variants relative to WT as determined by competition assay. Each symbol is a replicate competition assay, and exact *p*-values for the key comparisons are provided in the main text. (D) Mutation rate of indicated variants in mutations per nucleotide per strand copied as determined by Luria Delbruck fluctuation test. Each symbol is a replicate fluctuation test, and exact *p*-values for the key comparisons are provided in the main text. (E) In vitro kinetics of purified RdRp. Purified RdRp (2 μM), primer template (1 μM), and ATP were incubated, and samples were quenched at the indicated time points (schematic). The kinetics of complex assembly and single-nucleotide incorporation are expressed as μM extended template (y-axis) over time (x-axis). Representative data are shown. Complete data from replicates can be found in S1 Data 2E. All plotted data can be found in S1 Data. RdRp, RNA-dependent RNA polymerase; WT, wild-type.

If viral RdRp are constrained by a speed–fidelity trade-off, selection for increased replicative speed (r-selection) will increase mutation rate. We subjected the 3D^G64S-1nt^ point mutant (A6176G) to r-selection over serial passage by infecting cells at low multiplicity and harvesting progeny at 4.5 hours (midexponential phase of replication). The 3D^G64S^ point mutant reverted to WT within 15 passages in 5 independent lineages (Fig 2A). We only observed partial reversion at passage 15 in a subset of 24-hour control lineages, in which virus populations underwent twice as many cellular infection cycles per passage and experienced reduced r-selection. We next asked whether r-selection would lead to genetic compensation of the fidelity phenotype in 3D^G64S-3nt^, which has all 3 positions in the codon mutated to minimize reversion. After 50 passages of r-selection, we identified fixed and polymorphic single-nucleotide variants (SNVs) by next-generation sequencing of all r-selected and control (24-hour passage) populations of 3D^G64S^ and WT (Fig 2B).

Unbiased hierarchical clustering of SNVs by type and frequency indicates that the viruses explored distinct mutational pathways in adapting to either r-selective or control passaging regimes. Within the r-selected group, WT and 3D^G64S^ lineages clustered together, and we noted a larger number of SNVs within the coding region for the RdRp across the 5 3D^G64S^ populations. We found that a number of distinct SNVs increased viral fitness when introduced into the ancestral WT backbone. For example, the WT-VP4^S22G^ had a fitness of 2.62, and its presence in all r-selected and control lineages suggests that it mediates adaptation to HeLa cells (see SI, S1 Data 2C). In contrast, a mutation in the viral helicase found only in r-selected populations—2C^V127L^ (fitness 1.41–1.67 in WT and 1.11 ± 0.02 in 3D^G64S^, SI S1 Data 2C)—would be more likely to have a general effect on replicative speed.

To identify compensatory mutations, we focused our subsequent analysis on nonsynonymous mutations in the RdRp that were found predominantly in r-selected populations, shared among multiple lineages, and more frequent in 3D^G64S^ than in WT. Two mutations—U6261C/3D^I92T^ and A6813G/3D^K276R^—met these criteria, and their frequencies at passages 30 and 50 suggest that the I92T mutation may have arisen first. The 3D^I92T^ mutation, which was found in both r-selected WT (3 out of 5) and 3D^G64S^ (5 out of 5) lineages, did not change either fitness or mutation rate appreciably in the 3D^G64S^ background (Fig 2C and 2D). The r-selected K276R substitution, which was found in 3D^G64S^ lineages (4 out of 5) and not in WT populations, decreased overall fitness in both WT (0.92 ± 0.03; *p* = 0.0031 versus WT; *t* test) and 3D^G64S^ (0.55 ± 0.03; *p* = 0.005 versus 3D^G64S^; *t* test). It had no detectable effect on mutation rate in either background. The G64S/I92T/K276R triple mutant had a significant increase in fitness (0.7637; *p* = 0.0012 versus 3D^G64S^; *t* test) and mutation rate (8.71 × 10^−6^ s/n/r; *p* = 0.0120 versus 3D^G64S^; *t* test) compared to 3D^G64S^ and each double mutant. Therefore, direct selection for replicative speed led to indirect selection of mutations that together increase the poliovirus mutation rate, with sign epistasis among G64S, I92T, and K276R in the RdRp.

To gain mechanistic insight into the interactions among these 3 mutations, we analyzed the kinetics of single-nucleotide incorporation and misincorporation by purified RdRp. The 3D^G64S;I92T^ RdRp exhibits an assembly defect relative to 3D^I92T^ when incubated with purified primer template and ATP (Fig 2E and [6]). The K276R mutation partially compensates for this assembly defect in the 3D^G64S;I92T^ background, resulting in a 1.5- to 2-fold increase in incorporation of the correct nucleotide (A opposite U). This interaction is dependent on G64S because K276R reduced RdRp activity in the 3D^I92T^ background. While some poliovirus mutators exhibit altered kinetics of nucleotide misincorporation for G opposite U [30], the kinetics of 3D^G64S;I92T;K276R^ were similar to those of 3D^G64S;I92T^ (S1 Fig).

We further examined the relationship between RdRp speed and fidelity using a second poliovirus antimutator. The 3D^K359R^ RdRp has slower polymerization kinetics and higher fidelity relative to WT, and the 3D^K359H^ RdRp has similar characteristics [44]. In 2 separate experiments, we infected HeLa cells with 3D^K359H^ virus and recovered mutants after 1 or 2 passages. We observed 2 missense mutations in the 3D gene. Amino acid changes I331F and P356H were identified together in 1 experiment, and the P356H change was identified alone in the second. We introduced each of these amino acid substitutions alone or in combination into the 3D^K359H^ RdRp. In all cases, we observed an increase in the elongation rate (*k*pol) of the viral polymerase (a surrogate for speed) as well as the nucleotide misincorporation rate (*k*obs misincorporation for mutant relative to WT) in vitro (Table 1).

**Table 1:** Kinetic parameters for nucleotide incorporation and misincorporation for purified RdRp. The k_pol_ for the correct nucleotide measures the speed of polymerization in vitro. The k_pol,corr_/k_pol,incorr_ is an in vitro surrogate for fidelity, as it measures the relative rates of incorporation for the correct and incorrect nucleotides. A higher ratio indicates higher fidelity.

RdRp	Correct Nucleotide		Incorrect Nucleotide	
	k_pol_ (s^−1^)	K_d,app_ (μM)	k_pol_ (s^−1^) [× 10^−3^]^a^	k_pol,corr_/k_pol,incorr_
WT	37 ± 3	180 ± 40	4.2 ± 0.6	8,800
K359H	4.0 ± 0.2	250 ± 20	0.10 ± 0.02	40,000
I331F K359H	8.5 ± 0.1	170 ± 10	0.74 ± 0.13	11,000
P356S K359H	9.8 ± 0.3	110 ± 10	0.78 ± 0.09	13,000
I331F P356S K359H	17 ± 1	120 ± 10	4.9 ± 0.8	3,500

^a^Determined at saturating concentrations of nucleotide substrate.

Abbreviations: RdRp, RNA-dependent RNA polymerase; WT, wild-type.

**Fig 3:**
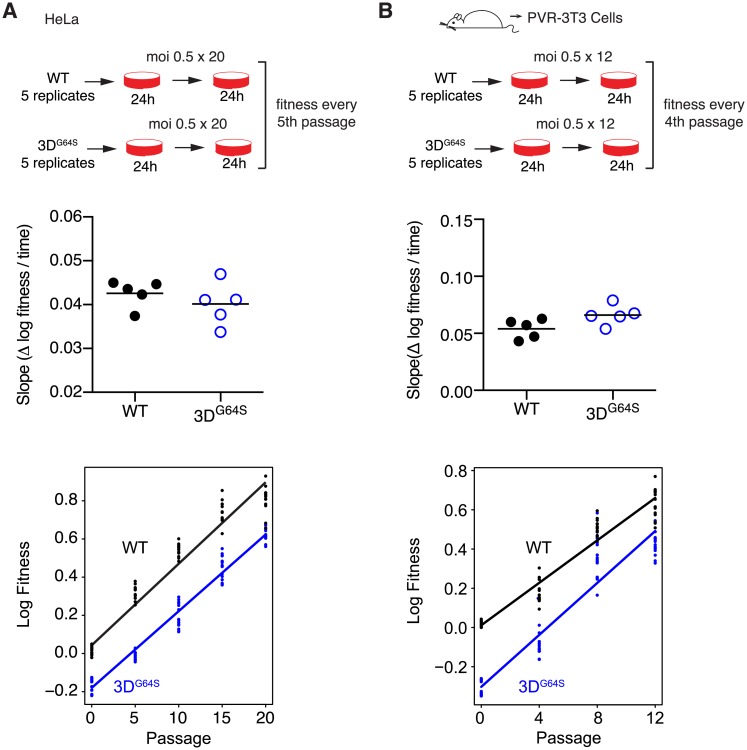
Adaptability of WT and 3D^G64S^ over 20 passages in HeLa (A) or 12 passages in a 3T3 cell line derived from mice transgenic for the PVR (B). Fitness values (≥3 replicate competition assays for each data point) were determined for populations from every fifth passage (panel A) or every fourth passage (panel B), and the adaptability in the top panels was expressed as the slope of the regression for log10 fitness over time for each of 5 independent lineages of WT (filled circles) and 3D^G64S^ (open circles, blue) for each cell line. The bottom panels show all the data from the 5 lineages together with the regression of log10 fitness over time. Exact *p*-values for the difference between the slopes for WT and 3D^G64S^ on HeLa (0.0129) and PVR-3T3 (0.0013) were derived from a mixed linear effects model (see Methods). All plotted data can be found in SI, S1 Data. HeLa, human epithelial cells; PVR, poliovirus receptor; WT, wild-type.

The adaptability of WT and high-fidelity viruses have generally been compared using assays that measure the acquisition of drug resistance, the reversion of an attenuating point mutation, or escape from microRNA in a limited number of replication cycles [5–7,34,36]. In these experiments, mutations come at little cost, and the assays essentially quantify the beneficial mutation rate. To capture better the impact of both deleterious and beneficial mutations on adaptability, we measured the fitness gain of WT and 3D^G64S^ over 20 passages in HeLa. While our WT strain is “culture-adapted,” we found that it was far from a fitness peak; both WT and 3D^G64S^ increased their fitness 10-fold in approximately 40 cellular infection cycles (20 passages, Fig 3A, S2 Fig). The difference in the rate of fitness gain between WT and 3D^G64S^ lineages was small but statistically significant (0.025 per passage, WT > 3D^G64S^; mixed linear effects model, *p* = 0.0129).

We examined adaptation to a completely distinct environment by repeating the experiment on our PVR-3T3 cell line [45,46]. In this alternative species and cell type, we actually observed greater fitness gain in the high-fidelity 3D^G64S^ variant relative to WT (0.121 per passage; mixed linear effects model, *p* = 0.0013). The larger fitness gain in 3T3 cells may reflect a larger supply of beneficial, compensatory mutations given the lower baseline fitness of 3D^G64S^ in these cells. These data suggest that, despite its 2-fold reduction in mutation rate, 3D^G64S^ is not mutation limited and that, in the absence of strong selection, any adaptive benefit of a higher mutation rate is countered by the fitness cost of increased mutational load (see Fig 1F and 1G and associated model above).

We next compared the phenotype of WT and 3D^G64S^ viruses in vivo, where the ability to generate genetic diversity may allow a virus to escape host immune restriction and to replicate better in a range of environments. The available data have suggested that the attenuation of 3D^G64S^ and other high-fidelity variants in experimental models is attributable to differences in the genetic diversity of the infecting population [7]. In this earlier work, viral diversity in the inoculum was manipulated using exogenous mutagen—2 passages in ribavirin and 5-fluorouracil followed by propagation in the absence of mutagen over 2 additional passages. The diversity of the mutant spectrum was assessed by Sanger sequencing of the 5’ noncoding and capsid-coding regions of viruses from 24 plaques.

Given the difficulty in controlling the type and level of genetic diversity in a population for correlative studies, we instead generated 5 independent stocks each of WT and 3D^G64S^ and compared their diversity through next-generation sequencing. Using an internally benchmarked analysis pipeline that dramatically reduces false positive variant calls (see [47] and Methods), we identified no variants at greater than 0.1% frequency. Therefore, we can exclude any significant differences in standing genetic diversity between our WT and 3D^G64S^ populations. Even at extremely high MOIs, variants present at <0.1% are unlikely to complement each other or to cooperate reproducibly in a cellular context or in vivo [48].

**Fig 4:**
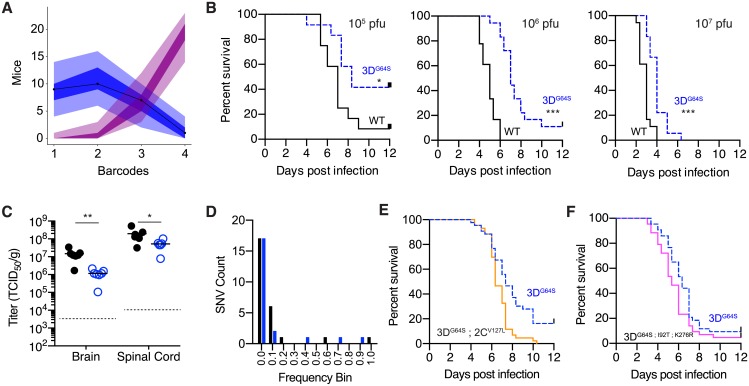
In vivo phenotype of WT and 3D^G64S^. (A) Maximum likelihood optimization of a simple binomial model (see S1 Text Model 2) estimated an average inoculum to CNS bottleneck size of 2.67 (lambda 2.44; 95% CI 1.39–3.82) based on experimental data for 4 barcoded poliovirus populations [49]. Shown are outputs of 10,000 simulations of the model (number of mice with 1, 2, 3, or 4 barcodes represented in the CNS). Each simulation represents 27 mice, and each mouse has a bottleneck size drawn from a zero-truncated Poisson with an average lambda of 2.43 (blue) or 10 (magenta). Line is actual data from [49], the shaded regions represent the area occupied by 95% of the simulations, and the dark shaded regions represent the interquartile range of the simulations. (B) Survival curves showing mice with paralysis-free survival over time for groups infected intramuscularly with 10^5^ pfu (left; *n* = 12 per virus), 10^6^ pfu (center; *n* = 18 per virus), and 10^7^ pfu (right; *n* = 18 per virus) of WT (black) or 3D^G64S^ (dashed blue). *p < 0.05; ***p < 0.001 by log rank test. (C) Viral titer in brain and spinal cord 5 days post intravenous inoculation with 10^7^ pfu of WT (filled circles) or 3D^G64S^ (open circles). *p < 0.05; **p < 0.005 by Mann Whitney U test; *n* = 7 mice in each group (out of 8 that were infected, 1 mouse in each group had titers below the limit of detection, dotted line). (D) Histogram of frequencies of intrahost SNVs identified in the spinal cords of 12 mice from panel C (7 infected with WT and 5 infected with 3D^G64S^). Black, synonymous or noncoding; blue, nonsynonymous. (E) Survival curves showing mice with paralysis-free survival over time for groups (*n* = 43 per virus combined from 2 experiments) infected intramuscularly with 10^6^ pfu of 3D^G64S^ (dashed blue) or 3D^G64S^;2C^V127L^ (orange). **p < 0.005 by log rank test; actual *p*-value 0.0012. (F) Survival curves showing mice with paralysis-free survival over time for groups (*n* = 43 per virus combined from 2 experiments) infected intramuscularly with 10^6^ pfu of 3D^G64S^ (dashed blue) or 3D^G64S;I92T;K276R^ (pink). *p < 0.05 by log rank test; actual *p*-value 0.0411. All plotted data can be found in S1 Data. CNS, central nervous system; SNV, single-nucleotide variant; WT, wild-type.

The absence of mutational diversity in our replicate WT and 3D^G64S^ stocks is important because poliovirus populations are subject to stringent bottleneck events, which further restrict intrahost diversity [49–51]. Work with barcoded RNA viruses suggests that the serial bottlenecks between the infecting population and the terminal population colonizing the central nervous system (CNS) are quite stringent [52,53], and we used published data to quantify the aggregate bottleneck size encountered by poliovirus in transgenic mice [46,49,50]. Maximum likelihood optimization of a simple probabilistic model estimated an aggregate bottleneck size of 2.67 between the inoculum and the CNS (Fig 4A, S1 Text Model 2). Therefore, the population that causes eventual disease in these mice is derived from no more than 2 to 3 viruses in the infecting population. In the setting of tight bottlenecks, many mutations will increase in frequency due to genetic drift as opposed to positive selection.

We infected groups of PVR mice intramuscularly with both WT and 3D^G64S^ populations. Both viruses were able to access the CNS efficiently through this route over a range of doses (Fig 4B, S2 Table), but there was a clear delay in the 3D^G64S^ group (*p* = 0.0239, *p <* 0.001, and *p <* 0.001 for 10^5^, 10^6^, and 10^7^ pfu inocula, respectively). This lag persisted even when we infected with doses 20-fold higher than the median lethal dose (LD_50_) of 3D^G64S^ (S3 Table). The difference in 3D^G64S^ attenuation compared to prior work [7] does not appear to be due to the mouse model used because other studies in cPVR mice have reported results similar to those presented here [36,54]. Both viruses spread to the CNS and replicated to high titers after intravenous inoculation, although WT titers in the brain and spinal cord were marginally higher at 5 days post infection (Fig 4C, *p* = 0.0012 for brain and *p* = 0.0221 for spinal cord, Mann Whitney U test). We characterized the mutations present in the CNS populations of 12 spinal cords of intravenously infected mice (Fig 4D, 7 WT and 5 3D^G64S^). Most mutations were rare, none were shared among mice, and there was an excess of synonymous or noncoding variants relative to nonsynonymous ones. These data are consistent with random sampling of the infecting population as opposed to positive selection.

We also examined the impact of the r-selected mutations on virulence. The 2C^V127L^ mutation, which conferred a fitness of 1.11 in the 3D^G64S^ background and does not appear to affect fidelity (S3 Fig), restored virulence to nearly WT levels (*p* = 0.0012, log rank test; compare 3D^G64S^ to 3D^G64S^;2C^V127L^ in Fig 4E). In contrast, the triple mutant, 3D^G64S;I92T;K276R^—which replicates with a WT mutation rate and a marginally increased fitness of 0.7637—was only slightly more virulent than the high-fidelity variant 3D^G64S^ (*p* = 0.0411, log rank test; compare 3D^G64S^ to 3D^G64S;I92T;K276R^ in Fig 4F). Therefore, restoration of replicative speed restored virulence in 3D^G64S^, but compensation of the fidelity phenotype did not.

## Discussion

We used a well-studied antimutator variant of poliovirus to identify the selective forces that optimize a pathogen’s mutation rate. Using 3 different assays, we identified a significant fitness cost to higher fidelity and directly link this cost to viral replication kinetics. Our quantitative model of the speed–fidelity trade-off suggests that selection for replicative speed has pushed viral mutation rates to a level that imposes a significant fitness cost at baseline due to lethal or highly deleterious mutations. Consistent with the trade-off model, direct selection for increased replicative speed led to indirect selection of polymerases with higher mutation rates. The genetic interactions are quite strong because the 2 compensatory mutations exhibited reciprocal sign epistasis. The speed–fidelity trade-off in the poliovirus RdRp appears to be a generalizable phenomenon because compensatory mutations that increased the replicative speed of the 3D^K359H^ antimutator also increased its mutation rate (Table 1). Given the structural similarity among viral RdRp, the polymerases of other RNA viruses are likely to be subject to the same speed–fidelity trade-off, and we predict that the molecular mechanisms governing polymerase kinetics and mutation rate will be similar.

Trade-offs are essentially constraints that force one parameter to change with another. In this case, viral mutation rate changes with replicative speed [55]. Similar trade-offs are central to the kinetic proofreading hypothesis, which posits a close relationship between the error rates of biosynthetic processes and the kinetics of their component reactions [56]. Studies of DNA replication and protein translation suggest that these systems optimize speed over accuracy as long as the error rates are within a tolerable range [57,58]. We find a similar phenomenon in viral RdRp, in which the WT generates an extraordinary amount of mutational load, largely because of the benefit in replicative speed.

Failure to consider evolutionary trade-offs can lead to teleological errors, in which the consequences of a process (e.g., increased genetic diversity) are misinterpreted as a cause (e.g., direct selection for a higher mutation rate [19,23,26]). Similarly, we find that the proposed link between within-host genetic diversity and virulence is confounded by the fact that faster replicating viruses are both more virulent and have higher mutation rates. The high mutation rates of RNA viruses and the highly deleterious fitness effects of mutations ensure that most genetic diversity is extremely rare and unlikely to be consistently maintained in the face of intrahost and interhost bottlenecks [53]. We do not dispute that virus populations will harbor minority variants, that a subset of these mutations may be adaptive or beneficial to the virus, and that some may be virulence determinants. However, the observation of genetic diversity is not in and of itself evidence that selection has optimized mutation rates for the future benefit of novel mutations. Indeed, our data show little adaptive benefit to a marginally increased mutation rate and identify no plausible mechanism whereby the observed increase in rare genetic diversity can influence pathogenesis. We suspect that RNA viruses are subject to other trade-offs of evolutionary significance, perhaps between polymerase speed and recombination rate or between recombination rate and polymerase fidelity. Here too it will be important to define the selective forces at play, thereby separating the causes from the consequences.

## Methods

### Ethics statement

The University of Michigan Institutional Animal Care and Use Committee reviewed and approved the protocols for all mouse studies described in this manuscript (Protocol ID PRO00008088). The University’s Animal Welfare Assurance Number on file with the NIH Office of Laboratory Animal Welfare (OLAW) is A3114-01. Information on humane endpoints used, the length of each experiment, the numbers of animals used and subsequently euthanized (S2 and S3 Tables), the frequency of monitoring, and animal welfare considerations are described below (“Infection of transgenic mice” section).

### Cells and viruses

A low passage stock of HeLa cells (<2 weeks in culture), previously obtained directly from ATCC (CCL-2), was kindly provided by Mary O’Riordan (University of Michigan). Except where noted, these cells were used for all experiments in this study and maintained in minimal essential media (MEM; Invitrogen 11090), supplemented with 10% fetal bovine serum (Gibco or Hyclone), 1x penicillin-streptomycin (Invitrogen 15140–148), 1x sodium pyruvate (Invitrogen 11360), 1x MEM alpha nonessential amino acids (Invitrogen 11140), and 1x glutamine (Invitrogen 25030). A second stock of HeLa of unknown passage history was obtained from Michael Imperiale (University of Michigan). These cells were only used for plaque assays to titer stocks and were maintained in Dulbecco’s modified Eagle’s media (DMEM; Invitrogen 11965) supplemented with 10% fetal bovine serum and 1x penicillin-streptomycin. PVR-3T3 cells are described below and were maintained in DMEM supplemented with 10% fetal bovine serum, 1x penicillin-streptomycin, and 1x glutamine. In all cases, cell lines were maintained for no more than 30 passages at a time. WT poliovirus and all mutants were generated from plasmid pEW-M, a Mahoney clone originally obtained from Eckard Wimmer (SUNY-Stonybrook) [59].

### Generation of PVR-3T3 cells

C57/BL6 PVR-Tg21 (PVR) mice [45,46] were obtained from S. Koike (Tokyo, Japan) via Julie Pfeiffer (UT Southwestern) and maintained in specific pathogen-free conditions. Primary MEFs were derived from PVR mice. Day 13.5 embryos were harvested and washed in phosphate-buffered saline (PBS). The heads and viscera were removed, and the body was minced with a sterile razor blade, trypsinized, and homogenized by pipetting with a 10 ml serological pipette. Cells were plated in DMEM supplemented with 10% fetal bovine serum, 1x penicillin-streptomycin, and 1x glutamine. An immortalized cell line was derived from PVR MEFs following the 3T3 protocol [60]. Briefly, freshly thawed MEFs were plated in 30 T25 flasks at a density of 3.8 × 10^5^ cells per flask in complete DMEM. Every third day, cells in each flask were trypsinized, counted, and transferred to fresh flasks at a density of 3.8 × 10^5^ cells per flask. As the cellular population began to increase (passages 13–15), cells were expanded into larger vessels and ultimately frozen down at passage 17.

### Site-directed mutagenesis

All mutations were introduced into either pEW-M or subclones using overlap extension PCR [61]. The presence of the desired mutation and the absence of additional mutations were verified by Sanger sequencing of the amplified insert and, in some cases, the entire genome.

### In vitro transcription, transfection, and viral stocks

Viral RNA was generated by in vitro transcription of the corresponding plasmid clone using T7 RNA polymerase, and virus was recovered following RNA transfection of HeLa. For transfections, 2.6 × 10^5^ HeLa were plated per well in a 12-well plate the day prior. One microgram of RNA was mixed with 4 μl TransIT mRNA transfection reagent (Mirus 2225) and 100 μl OptiMEM (Invitrogen 31985), incubated according to the manufacturer’s protocol and applied to cells. Passage 0 virus was harvested at 100% CPE (within 24–48 hours). Passage 1 stocks were generated by passaging 100 μl of passage 0 virus on fresh cells and were titered by either plaque assay or TCID_50_. Passage 2 and 3 stocks were generated by passaging at an MOI of 0.01. For all stocks, cells were subjected to 3 freeze–thaw cycles and the supernatants clarified by centrifugation a 1,400 × g for 4 minutes. These supernatants were stored at −80 °C in aliquots to limit the number of subsequent freeze–thaw cycles.

### Competition assay for viral fitness

Competition assays were performed essentially as described in [17,42]. For the experiment in Fig 1A, HeLa cells were plated in 12-well plates, at a density of 2.6 × 10^5^ per well the day prior to infection. Cells were infected at a total MOI of 0.1 with an equal TCID_50_ of WT and 3D^G64S^. Three replicate wells were infected with each pair of viruses in 250 μl for 1 hour with occasional rocking. After 1 hour, the inoculum was removed and 1 ml fresh media applied. Passage 1 virus was harvested after an additional 7 hours (8 hours since infection). The titer of the passage 1 virus was used to calculate the dilution factor necessary to maintain an MOI of 0.1 for the subsequent 5 passages. RNA was harvested from each passage using Trizol (Ambion 15596026). Random hexamers were used to prime cDNA synthesis with 1/10 of the RNA. Each cDNA was analyzed by qRT-PCR using 3 different primer and/or probe sets with duplicate PCR reactions for each sample and primer set. The first set—COM2F 5’ CATGGCAGCCCCGGAACAGG 3'‘ and COM2R 5’ TGTGATGGATCCGGGGGTAGCG 3’—was used to quantify total viral genomic RNA in an SYBR green reaction (Power SYBR Green PCR Master Mix; Thermo 4368708). Two custom TaqMan probes (Applied Biosystems) were used to quantify the number of WT and 3D^G64S^ genomes. Duplicate wells were averaged, and relative amounts of WT and 3D^G64S^ RNA were determined by normalizing the cycle thresholds for each of these probes to those of the COM primer set (ΔCt = Ct_Virus_ − Ct_COM_). The normalized values for virus passages 1–6 were then compared to passage 0 to obtain a ratio relative to P0 (ΔΔCt = ΔCt_PX_ − ΔCt_P0_). This relative Ct value was converted to reflect the fold change in the ratio (Δratio = 2^−ΔΔCt^). The change in ratio of the mutant relative to the change in ratio of the WT as a function of passage is the fitness ([Δlog ratio_Mut_ − Δlog ratio_WT_]/time). Competition assays in ribavirin (Fig 1F) were performed in the exact same manner except that serum-free media were used in both drug and mock passages.

For all other competition assays (Fig 3), we compared the experimental virus (e.g., WT P4, 3D^G64S^ P8, etc.) to a tagged WT reference (Tag8). We plated 2.6 × 10^5^ cells per well (either HeLa or PVR-3T3) in 12-well plates. Infections were performed at an MOI of 0.05 in 250 μl complete media for 1 hour. After an hour, the media were aspirated and fresh 1 ml growth media applied. All passages were for 24 hours. The dilution factor between passages required to maintain this MOI was 400 for HeLa competitions and 350 for PVR-3T3 competitions. All RNA harvests for these competitions were performed in 96-well plates using Purelink Pro 96 Viral RNA/DNA kits (Invitrogen 12280), and cDNA synthesis was performed as above. In addition to the COM primer set (see above), we used primer pairs Tag8 seq.tag 5’ TTCAGCGTCAGGTTGTTGA 3’ + Rev. WT seq.tag 5’ CAGTGTTTGGGAGAGCGTCT 3’ and WT seq.tag 5’ AGCGTGCGCTTGTTGCGA 3’ + Rev. WT seq.tag 5’ CAGTGTTTGGGAGAGCGTCT 3’ to quantify the Tag8 reference and test samples, respectively. Note also that in these competitions the regressions were fit through passages 1–4 and excluded P0 as slight deviations from a 1:1 ratio of the two viruses in the inoculum can skew the slope when fit through this data point.

### Plaque-size assay

Plaque assays were performed on subconfluent monolayers (7.5 × 10^6^ on day of infection) in 10 cm dishes. The amount of virus applied to each plate was determined empirically to ensure well-spaced plaques (approximately 30 per 10 cm dish). Plates were stained with crystal violet at 72 hours post infection. Each plate was scanned individually at 300 dpi using a flat-bed scanner. Sixteen-bit image files were analyzed using ImageJ. Brightness, contrast, and circularity thresholds for plaque identification were set using uninfected plates.

### Single replication cycle growth curve

The day prior to infection, 4 × 10^5^ HeLa cells were plated in 12-well plates with 45 wells per virus (9 time points and 5 replicates per time point). Cells were infected at an MOI of 1 in 150 μl volume, and the infections were synchronized by incubation on ice for 1 hour with occasional rocking. At 1 hour, the inocula were aspirated, each well was washed twice with ice-cold PBS, and 1 ml of fresh, prewarmed growth media were applied to all wells. One set of 5 wells was immediately frozen as the t = 0 hours time point. All other plates were returned to the incubator, and a set of 5 wells was removed and frozen at t = 1.5, 2, 2.5, 3, 3.5, 4, 5, and 7 hours. All samples were titered by TCID_50_. The growth curve on PVR-3T3 cells was performed using a similar protocol, except that 5 × 10^5^ cells were plated the day prior and the time points were t = 1, 2, 3, 4, 5, 6, 7, and 8 hours.

### Measurement of viral mutation rates

Mutation rates were measured by Luria-Delbruck fluctuation test, which in this case quantifies the rate at which the poliovirus 2C protein acquires the necessary point mutations to permit viral growth in 1 mM guanidine hydrochloride [62–64]. Each fluctuation test was performed with 29 replicate cultures in 48-well plates. A total of 65,000 HeLa cells per well were plated the day prior to infection. In all cases, the media were changed to serum-free media 3 hours prior to infection. For infections in ribavirin, this serum-free media also included drug at the specified concentrations. Each well was infected in 200 μl volume with 1,000 to 4,000 pfu per well depending on the virus and experimental condition. Five independent aliquots were also saved for subsequent titering (see N_i_ below). For infections in ribavirin, the infection media also included drug at the specified concentrations. Infected cells were incubated for 7 hours and then frozen. The lysed cells and media were harvested following 3 complete freeze–thaw cycles and transferred to a microcentrifuge tube. The empty wells were rinsed with 300 μl of complete growth media and combined with the initial 200 μl lysate. This 500 μl lysate was clarified by centrifugation at 1,400 × g for 4 minutes. Twenty-four wells were titered by plaque assay with 1 mM guanidine hydrochloride in the overlay (see P_0_ below). Five wells were titered by standard plaque assay without guanidine hydrochloride (see N_f_ below). The mutation rate, μ_0_, was estimated from these data using the P_0_ null-class model: μ_0_ = −lnP_0_/(N_f_-N_i_), where P_0_ was the fraction of the cultures that yielded no guanidine-resistant plaques, N_f_ was the average number of pfu in the absence of guanidine, and N_i_ was the average number of pfu in the inoculum. As described in [65], μ_0_ can be converted to the mutation rate in nucleotide units by correcting for the mutation target (number of mutations leading to the scored phenotype, T) and the number of possible mutations at each target site (constant, 3) using the equation μ = 3μ_0_/T. The number of distinct mutations that could yield the guanidine-resistant phenotype was determined empirically by isolating and sequencing the entire 2C open reading frame for 15 guanidine-resistant plaques derived from WT virus and 15 guanidine-resistant plaques derived from WT virus treated with 200 μM ribavirin. In each case, we found 6 mutations that mediated resistance, although there were 7 total among 30 plaques (S1 Table).

### Mutagen sensitivity assay

HeLa cells were plated the day prior to infection at a density of 2.6 × 10^5^ cells per well in a 12-well dish. On the day of infection, monolayers were pretreated with 0 to 600 μM ribavirin in serum-free media for 3 hours, then infected with virus at an MOI of 0.1 (50,000 pfu) for 60 minutes. The cells were washed once in PBS and incubated in ribavirin for an additional 24 hours. Viral supernatants were harvested by freeze–thaw as above and titered by tissue culture infectious dose.

### R-selection through serial passage

For each passage, HeLa cells were plated the day prior to infection in 6-well plates at a density of 7.25 × 10^5^ cells per well, yielding 1.2 × 10^6^ cells on the day of infection. Infections were initiated with passage 3 stocks of either WT or 3D^G64S^, and each passage was performed at an MOI of 0.5 (6 × 10^5^ TCID_50_ units) in 1 ml of media for 1 hour with occasional rocking. After 1 hour, the inoculum was aspirated, the cells were washed twice with PBS, and 2 ml of fresh growth media were applied. For the first 15 passages, WT and 3D^G64S^ viruses were harvested at 4 and 4.5 hours, respectively. For passages 16 through 50, WT and 3D^G64S^ viruses were harvested at 3.5 and 4 hours, respectively. Control populations were infected in the same manner except that viruses were harvested at 24 hours post infection. There were 5 r-selected WT lineages, 5 r-selected 3D^G64S^ lineages, 5 control WT lineages, and 5 control 3D^G64S^ lineages. Viruses were titered at every fifth passage to maintain an MOI of approximately 0.5.

### Selection and identification of second-site suppressors of RdRp variant 3D^K359H^

HeLa cells were transfected by electroporation with viral RNA transcript, added to HeLa cell monolayers, and incubated at 37 °C. After 2 days, the media were passaged onto a separate monolayer of HeLa cells. Upon cytopathic effect, viruses were harvested by 3 repeated freeze–thaw cycles, cell debris was removed by centrifugation, and viral supernatants were titrated. In this time frame, the titer increased approximately 40-fold (from 5.1 × 10^5^ pfu/mL to 2.1 × 10^7^). Viral RNA was isolated with QIAamp viral RNA purification kits (Qiagen) according to the manufacturer’s instructions. The 3Dpol cDNA was prepared from purified viral RNA by RT-PCR and sequenced. The I331F and P356S substitutions were identified together in 1 experiment, and the P356S substitution was identified in a second.

### In vitro assays of RdRp function

All mutations were introduced into the pET26Ub-PV 3D [66] or pSUMO-PV-3D [67] bacterial expression plasmids using either overlap extension PCR or QuickChange Site-Directed Mutagenesis. The presence of the desired mutations and the absence of additional mutations were verified by DNA sequencing. PV 3Dpol RdRps were expressed and purified as described previously [66,67]. The sym-sub assays used to measure assembly and elongation kinetics of purified RdRp on a defined template were performed as described in [6,30,68]. All assays had 1 μM primer/template and 2 μM enzyme.

### Adaptability of WT and 3D^G64S^

For HeLa cells, adaptability was measured using the 24-hour passage control lineages from the r-selection experiment. The fitness values of WT and 3D^G64S^ populations were measured by competition assay, as above, using samples from passages 0, 5, 10, 15, and 20. For PVR-3T3 cells, serial passages were performed as follows. Cells were seeded in 6-well plates at a density of 7.6 × 10^5^ cells per well the day prior to infection, yielding approximately 1 × 10^6^ the day of infection. Serial passage lineages were initiated with passage 1 stocks of either WT or 3D^G64S^, and each passage was performed at an MOI of 0.5 in 1 ml for 1 hour. After 1 hour, the inoculum was aspirated, the cells were washed twice with PBS, and 2 ml of fresh growth media were applied. Viruses were harvested at 24 hours and titered every fourth passage to ensure an MOI of 0.5. There were 5 replicate lineages of WT and 3D^G64S^.

### Infection of transgenic mice

Six- to 9-week-old mice were used for all experiments. The age range and distribution of males and females in each group for each experiment are reported in S2 Table. On the day of each infection, a general health exam was performed on all animals by university veterinary technical staff, and animals were assigned unique ear tag identifiers. Study animals were housed in BSL2 conditions. Females were housed together by group. Males from the same litter were housed together and were separated by group as needed. Enrichment was provided when any animal became single-housed due to fighting or other indication. Animals were fed 5LOD rodent chow. Because hindlimb paralysis was an expected outcome of this study, moist chow placed on floor of cage and/or diet gel was provided as a supplement. Water was provided ad libitum. Temperature and humidity were monitored and recorded daily by husbandry staff on general housing room sheets. Alternating 12-hour light and dark cycles were in place as per standard housing.

For survival analyses, mice were infected intramuscularly with 50 μl to each hindlimb for the total dose of 100 μl. Mice were observed twice daily for lethargy, hunched posture, scruffy fur, paralysis, or decreased mobility and were euthanized when they exhibited bilateral hindlimb paralysis. Over 90% of all assessments were performed by members of the university veterinary technical staff, who were blinded to the hypotheses and expected outcomes of the studies. All surviving animals were euthanized after 12 days. These endpoints were also used to calculate the PD50 using the Spearman-Karber method (S3 Table).

For tissue distribution analyses, mice were infected intravenously via tail vein with 100 μl and observed twice daily as above. Mice were euthanized for severe morbidity (as above) or on day 5, the conclusion of the experiment. Whole organs were isolated from all mice and homogenized in PBS using a Bead Beater. The homogenates were clarified by centrifugation at 15,800 × g for 4 minutes in a microfuge and the supernatant extracted with chloroform. Half of this supernatant was titered by TCID_50_. RNA was extracted from the remainder using Trizol.

### Next-generation sequencing

We amplified poliovirus genomes as 4 overlapping cDNA by RT-PCR. RNA was harvested from either cell-free supernatants or tissues as above and was reverse-transcribed using the SuperScript III First Strand Synthesis System for RT-PCR (Invitrogen 18080) and a mixture of random hexamers and oligo dT primer. The 4 genomic fragments were amplified using primer pairs: WFP37 FORWARD BASE 1 5’ TTAAAACAGCTCTGGGGTTGTACC 3’ + WFP41 REVERSE BASE 2434 5’ GCGCACGCTGAAGTCATTACACG 3’; WFP39 FORWARD BASE. 1911 5’ TCGACACCATGATTCCCTTTGACT 3’ + WFP42 REVERSE BASE 4348 5' AATTTCCTGGTGTTCCTGACTA 3'; WFP13 FORWARD BASE 4087 5' ATGCGATGTTCTGGAGATACCTTA 3' + WFP43 REVERSE BASE 5954 5' CCGCTGCAAACCCGTGTGA 3'; WFP40 FORWARD BASE 5545 5' TTTACCAACCCACGCTTCACCTG 3' + WFP33 REVERSE BASE 7441 5' CTCCGAATTAAAGAAAAATTTACCCC 3'. The thermocycler protocol was 98 °C for 30 seconds, then 30 cycles of 98 °C for 10 seconds, 68 °C for 20 seconds, and 72 °C for 3 minutes, followed by a single cycle of 72 °C for 5 minutes, then 4 °C hold. For each sample, amplification of all 4 fragments was confirmed by gel electrophoresis, and equal quantities of each PCR product were pooled. Seven hundred and fifty nanograms of each cDNA mixture were sheared to an average size of 300 to 400 bp using a Covaris S220 focused ultrasonicator. Sequencing libraries were prepared using the NEBNext Ultra DNA library prep kit (NEB E7370L), Agencourt AMPure XP beads (Beckman Coulter A63881), and NEBNext multiplex oligonucleotides for Illumina (NEB E7600S). The final concentration of each barcoded library was determined by Quanti PicoGreen dsDNA quantification (ThermoFisher Scientific), and equal nanomolar concentrations were pooled. Residual primer dimers were removed by gel isolation of a 300 to 500 bp band, which was purified using a GeneJet Gel Extraction Kit (ThermoFisher Scientific). Purified library pools were sequenced on an Illumina MiSeq with 2 × 250 nucleotide paired-end reads. All raw sequence data have been deposited at the NCBI short-read archive (Bioproject PRJNA396051, SRP113717).

### Variant detection

Sequencing reads that passed standard Illumina quality-control filters were binned by index and aligned to the reference genome using bowtie [69]. SNVs were identified and analyzed using DeepSNV [70], which relies on a clonal control to estimate the local error rate within a given sequence context and to identify strand bias in base calling. The clonal control was a library prepared in an identical fashion from the pEW-M plasmid and was sequenced in the same flow cell to control for batch effects. True positive SNVs were identified from the raw output tables by applying the following filtering criteria in R: (i) Bonferonni-corrected *p* < 0.01, (ii) average MapQ score on variant reads >20, (iii) average phred score on variant positions >35, (iv) average position of variant call on a read >62 and <188, and (v) variant frequency >0.001. We only considered SNVs identified in a single RT-PCR reaction and sequencing library for samples with copy number ≥10^5^ genomes/μl supernatant or in 2 separate RT-PCR reactions and sequencing libraries for samples with copy number 10^3^ to 10^5^ genomes per μl (e.g., in tissue studies). Our strategy for variant calling as well as our benchmarked sensitivity and specificity are described in [47], and all code can be found at https://github.com/lauringlab/variant_pipeline.

### Statistical analysis

No explicit power analyses were used in designing the experiments. In most cases, we used 5 biological replicates. In a few cases, we used fewer (3) or more (7) where the variance was either sufficiently low or high. The number of replicates, the statistical tests used, and the relevant *p*-values are reported in each figure legend or the main text (Fig 2C and 2D only). All replicates within the dynamic range of each assay are reported (i.e., no replicate experiments were excluded). Data on the relative adaptability of WT and 3D^G64S^ populations were analyzed with a 3-level linear mixed-effects model estimating a random slope and intercept of time nested within each fitness measurement replicate (measID), nested within each lineage replicate (repID). Virus was included as a fixed effect. Models were fit with the R package lme4; all code for this model can be found at https://github.com/lauringlab/speed_fidelity.

## Supporting information

S1 Data Plotted data for all figures. (XLSX)

S1 Text Models of the speed–fidelity trade-off (model 1) and within-host bottlenecks (model 2). (DOCX)

S1 Fig In vitro assay of polymerase-mediated single-nucleotide incorporation. (PDF)

S2 Fig Plots of fitness versus passage in adaptability experiment. (PDF)

S3 Fig Mutagen sensitivity of 2C-V127L variants. (PDF)

S1 Table Mutations conferring resistance to 1 mM guanidine. (DOCX)

S2 Table Number, age, and sex of mice used in all experiments. (DOCX)

S3 Table Raw data for calculation of LD_50_. LD_50_, median lethal dose. (DOCX)

## Abbreviations

CNS: central nervous system
DMEM: Dulbecco’s modified Eagle’s media
HeLa: human epithelial cells
LD_50_: median lethal dose
MEF: mouse embryonic fibroblast
MEM: minimal essential media
MOI: multiplicity of infection
PBS: phosphate-buffered saline
PVR: poliovirus receptor
qRT-PCR: quantitative reverse transcription polymerase chain reaction
RdRp: RNA-dependent RNA polymerase
SNV: single-nucleotide variant
WT: wild-type
