# Supplementary material for: A speed–fidelity trade-off determines the mutation rate and virulence of an RNA virus
Source: PLoS Biol. 2018 Jun 28;16(6):e2006459. doi: 10.1371/journal.pbio.2006459 (PMC6040757; doi:10.1371/journal.pbio.2006459)
Supplement: S1 Text — (DOCX) [file pbio.2006459.s002.docx]

**Supporting Information Model 1 – Speed fidelity trade off**

The premise of the speed-fidelity tradeoff is that the outcome of competition between two viral strains will be determined by two opposing forces – the speed with which the genome can be replicated and the error rate of replication. The faster genome replication happens, the more errors that occur and the greater the mutational load. In this scenario an optimal competitive fitness will be achieved exactly where the increase in fitness is counterbalanced by decrease in fitness from excess mutational load. Here we present a simple mathematical model to demonstrate this tradeoff, and fit the model to the experimental data.

We start with the classical estimation by Haldane (1937) of the equilibrium mean population fitness, w, as a function of the genomic deleterious mutation rate (U_d_) in units of deleterious mutations per genome per generation.

w=e^−Ud^

The relationship is shown graphically, here.


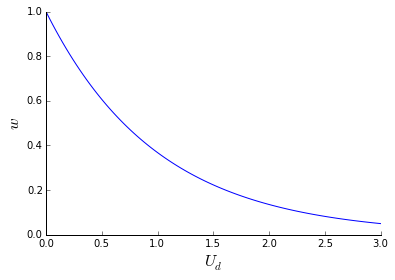


Now we consider competition between two strains that differ in both their speed, c, and their fidelity (as manifest by a deleterious mutation rate, U). If you have two strains, a and b, then the relative fitness of a to b will be:

*w_a,b_=(c_a_e^−Ua^)/ (c_b_e^−Ub^)*

Where c_a_ and c_b_ are the genome replication rates and U_a_ and U_b_ are the deleterious genomic mutation rates per genome per generation for strains a and b, respectively.

To understand the effect of a mutagen on the relative fitness of one strain to another, we add a mutation rate multiplier (mu external, σ), which multiplicatively modifies the baseline mutation rate. We constrain σ to take values greater than one.

w_a,b_=(c_a_e^−Ua∗σ^)/(c_b_e^−Ub∗σ^)

As shown below, as the mutation rate multiplier goes up, the fitness of a high fidelity variant increases relative to wild type (WT). The plot on the left shows both strains (WT and a high fidelity variant together). The equilibrium, where the two strains have equal fitness, is at a sigma value where the two lines cross (as indicated by the arrow, right)

With this simple model, we can use the empirical estimates of mutation rates and relative fitness values over a range of ribavirin concentrations (which increase the mutation rate multiplier, σ) to estimate the deleterious mutation rate and the amount of mutation load experienced by WT poliovirus and the 3D^G64S^ high fidelity variant. The data are presented in Figures 1F and 1G and available in the annotated Jupyter notebook available at https://github.com/lauringlab/speed_fidelity.


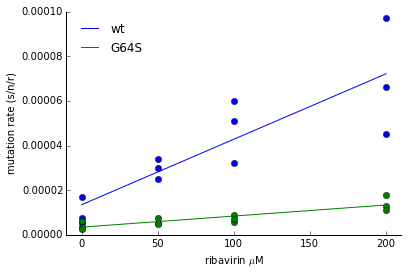


The G64S mutation has two effects (see also Fig. 1G). The mutation results in an increase in fidelity, represented as a downward shift in the line. The mutation also results in relative resistance to ribavirin, which manifests as a decreased slope. Both of these need to be included to estimate the mutational load experienced by WT and the 3D^G64S^ mutant replicating in the presence of increasing mutagen. Linear regression fit to both the WT and 3D^G64S^ mutation rate produce a good fit (r^2^ of .73 and .76 respectively) that is highly statistically significant (p < 0.001 for both). The fit of the linear regression was used to estimate the mutation rate for WT and 3D^G64S^ in the absence of ribavirin (1.34 x 10^-5^ s/n/r and 3.43 x10^-6^ s/n/r respectively) and at the point of equilibrium.

We solved for the two unknown variables: (i) *n,* the number of deleterious sites in the genome, and (ii) *c,* the fitness cost of the G64S mutation in the absence of mutational load, which is relative to the wild type (arbitrarily set to 1). Because there are two unknown variables, we need two equations to solve for them. We use the measured relative fitness in the absence of ribavirin and the ribavirin concentration where the relative fitness is expected to be 1 (150µM, see Fig. 1F). We used the mutation rates estimated for each strain and ribavirin concentration (μstrain,conc). At 0µM ribavirin, the fitness of 3D^G64S^ relative to WT was measured as 0.67, which gives us our first equation:

c∗e^−μG64S,0∗n^ / e^−μwt,0∗n^ = 0.67

Next, we looked at the competitive fitness data and used the point at which the two strains have equal fitness (approximately 150μM of ribavirin, see Fig. 1F):

c∗e^−μG64S,150∗n^ / e^−μwt,150∗n^ = 1.0

Now, with two equations, we solved for the two unknown values (n and c). Assuming they have equal fitness at 150µM, the fitness cost of 3D^G64S^ absent the cost from mutational load is 0.60. The effective number of sites with deleterious mutations is 10959 (48% of all possible mutations, given 3 possibilities at every site). The fitness cost due to mutation load that is experienced by WT in absence of ribavirin is 0.137. The fitness cost due to mutational load experienced by 3D^G64S^ in absence of ribavirin is 0.037. These relationships are shown graphically below, where the dashed line indicates the fitness in the absence of mutational load, the shaded area indicates the effect of mutational load on fitness and the solid lines indicate the overall effect on fitness.


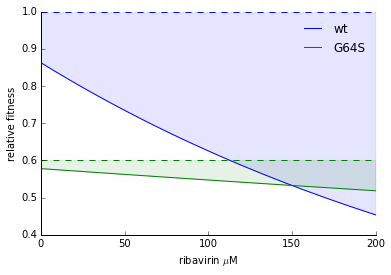


**Supporting Information Model 2 – Within host bottleneck**

We developed the following model to measure the effective bottleneck that restricts poliovirus populations between the site of inoculation and the central nervous system. We applied a simple probabilistic model to data described in [48]. In this study, 27 mice were infected with 2 x 10^7^ PFU of poliovirus (2-5 fold higher than the LD_50_ in this particular mouse model). The inocula consisted of 4 subpopulations at equal concentrations, each tagged with a neutral sequence bar code. In separate experiments, the authors showed that all 4 bar codes were present at the site of infection and that all four bar codes were capable of replicating simultaneously in the brain. Rarely were all 4 bar codes present in the brain following infection, suggesting that the populations were subject to within host bottlenecks. Similar results were observed for IV and IP routes of infection. In fact, IM appeared to be the least stringent mode of infection. To estimate the bottleneck between the site of infection and the brain, we modeled the infection process as a random sampling event. This assumption was justified as: (i) there is no evidence that a "jackpot" mutation is needed to enter the central nervous system. (ii) the bar codes were selectively neutral. (iii) all bar codes were equally likely to be present in the brain.

The probability of a sample size of $n$ containing $K$ unique types given there are $N$ total unique types available (all present at equal frequency) derives from discrete probability theory and is given by

$$P(K|N,n)=\binom{N}{k}(\frac{k}{N})^{n}[1-\sum_{i=1}^{k-1} \binom{k}{i}(\frac{k-i}{k})^{n}(-1)^{i+1}]$$

(see for example, Ross SM. 2010. *A First Course in Probability*. Prentice Hall, pages 121-122). In terms of the above experimental design, we are interested in the probability a subset of size $n$ containing 1,2,3, or 4 barcodes ($K$) given 4 possible barcodes ($N$).

Maximum likelihood optimization revealed that a bottleneck of 4 PFU best matched the data. However, this model was constrained, in part, by the fact that no smaller bottleneck could account for the presence of 4 barcodes in the CNS even though this rarely occurred. Indeed simulations revealed this model predicted a higher average number of barcodes than experimentally observed. In particular the model underestimated the probability of only 1 barcode infecting the CNS.

To account for experimental variability in bottleneck sizes between mice we allowed the bottleneck to vary according to a zero-truncated Poisson distribution. We truncated the Poisson distribution because at the high doses used in the original experiment and replicated in this current work poliovirus entered the CNS in all infected mice. That is, there were no mice with zero barcodes.

When we allow $n$, the bottleneck size, and in our model, to follow a zero-truncated Poisson distribution parameterized with λ. The likelihood of observing $k$ barcodes given $\lambda$ is

$$L(\lambda)=P(K|\lambda)=\sum^{n} P(K|N,n)P(n|\lambda)$$

Where $P(k|N,n)$ is our expression above and $P(n|\lambda)=\frac{\lambda^{n}}{(e^{n}-1)n!}$ or the probability of $n$ in a zero-truncated Poisson with a parameter $\lambda$. We approximated the infinite sum above with a partial sum of the first 100 terms as we expected a small bottleneck, and the probability of an $n$ of 50 with $\lambda=100$ is on the order of ${10}^{-10}$ and negligible. We then searched for the $\lambda$ that maximized the sum of the log of this likelihood, which was calculated for each mouse. We found that a $\lambda$ of 2.44 with a 95% confidence interval of (1.39 - 3.82) best fit the data. The mean of a zero-truncated Poisson is given by $\frac{\lambda e^{\lambda}}{e^{\lambda}-1}$. Therefore the mean bottleneck size is 2.67.


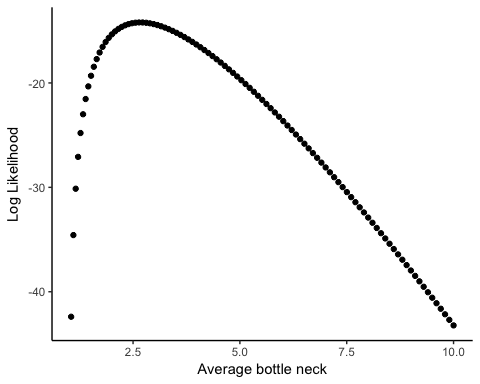


To test the fit we performed 10,000 simulations. Each simulation included 27 mice and each mouse had a bottleneck size drawn from a zero-truncated Poisson with a $\lambda$ of 2.44. For illustration we also simulated the data with an average bottleneck of 10. The output of the simulations is shown below and in Fig. 4A. The shaded regions represent the area occupied by 95% of the simulations with the dark regions representing the interquartile range of the simulations.


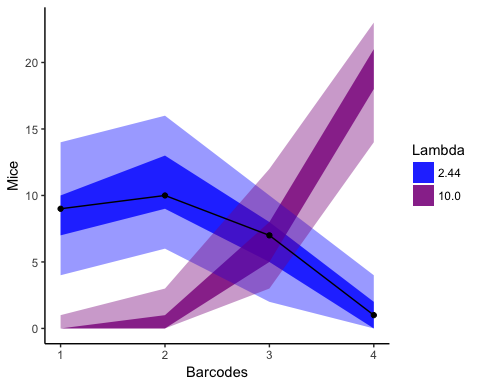


We also checked the fit by asking how the output of the simulations matched the actual experimental data (e.g. how often did we see 9 mice with 1 bar code, 10 with 2, 7 with 3, and 1 with 4).


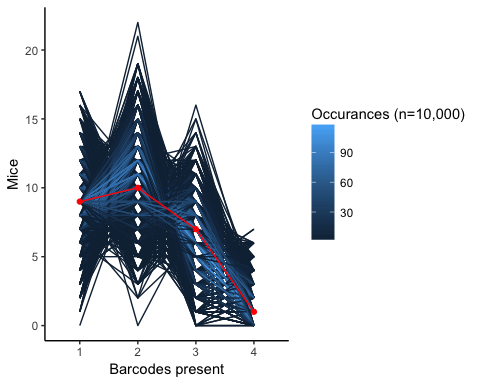


In contrast, we did not replicate the data once in 10,000 simulations with a bottleneck of 10.


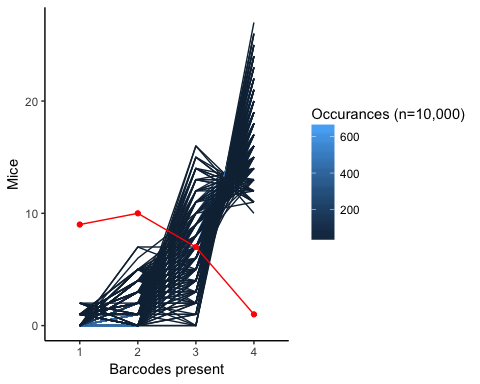


While we modeled the infectious process from inoculation to invasion of the CNS as a single sampling procedure, this process is made up of several bottlenecks imposed on the virus as it passes through different body compartments [49]. We, therefore, interpret our mean bottleneck of 2.67 as an aggregate, within-host bottleneck.

Population bottlenecks have been show to be dose dependent. However, it is likely that the inoculating dose modeled here, and used in the presented work, saturates this dose dependency. For example, Pfeiffer et al. only observed a slight increase in the average number of barcodes present in the CNS when mice were inoculated with 100x more PFU. In fact, the bottleneck appears to only be overcome when very young (two week old) mice were inoculated.
