# Supplementary material for: A speed–fidelity trade-off determines the mutation rate and virulence of an RNA virus
Source: PLoS Biol. 2018 Jun 28;16(6):e2006459. doi: 10.1371/journal.pbio.2006459 (PMC6040757; doi:10.1371/journal.pbio.2006459)
Supplement: S1 Fig — (PDF) [file pbio.2006459.s003.pdf]

Figure S1

A

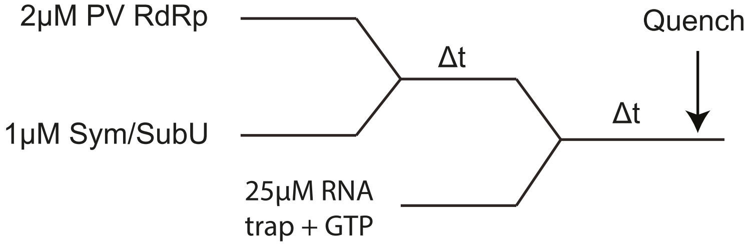

B

	GMP misincorporation	
Enzyme	*k*pol (s^{-1})	*K*d,app (μM)
WT	1.5 ± 0.1 x10^{-2}	250 ± 10
K276R	5.0 ± 0.1 x10^{-3}	250 ± 80
G64S-K276R	2.0 ± 0.1 x10^{-3}	300 ± 50
G64S	4.0 ± 0.1 x10^{-3}	300 ± 40

S1 Figure. In vitro assay of polymerase mediated single nucleotide incorporation. (A) Schematic of GTP misincorporation assay (G opposite the U). Primer-template (sym-subU) and polymerase are assembled in the absence of nucleotide. GTP and a 25-fold excess of unlabeled trap RNA are then added after an incubation period (Δt). Excess of RNA trap ensures that if the polymerase dissociates from primer-template it is taken up by the trap and cannot re-assemble. (B) Kpol and Kd for GMP misincorporation. Concentrations of GTP were used over time-points to calculate Kpol and Kd. Each GTP time-course was plotted to single exponential, then combined plot to hyperbola. Note that all polymerase variants have the I92T mutation.
