# Supplementary material for: A speed–fidelity trade-off determines the mutation rate and virulence of an RNA virus
Source: PLoS Biol. 2018 Jun 28;16(6):e2006459. doi: 10.1371/journal.pbio.2006459 (PMC6040757; doi:10.1371/journal.pbio.2006459)
Supplement: S2 Fig — (PDF) [file pbio.2006459.s004.pdf]

Figure S2

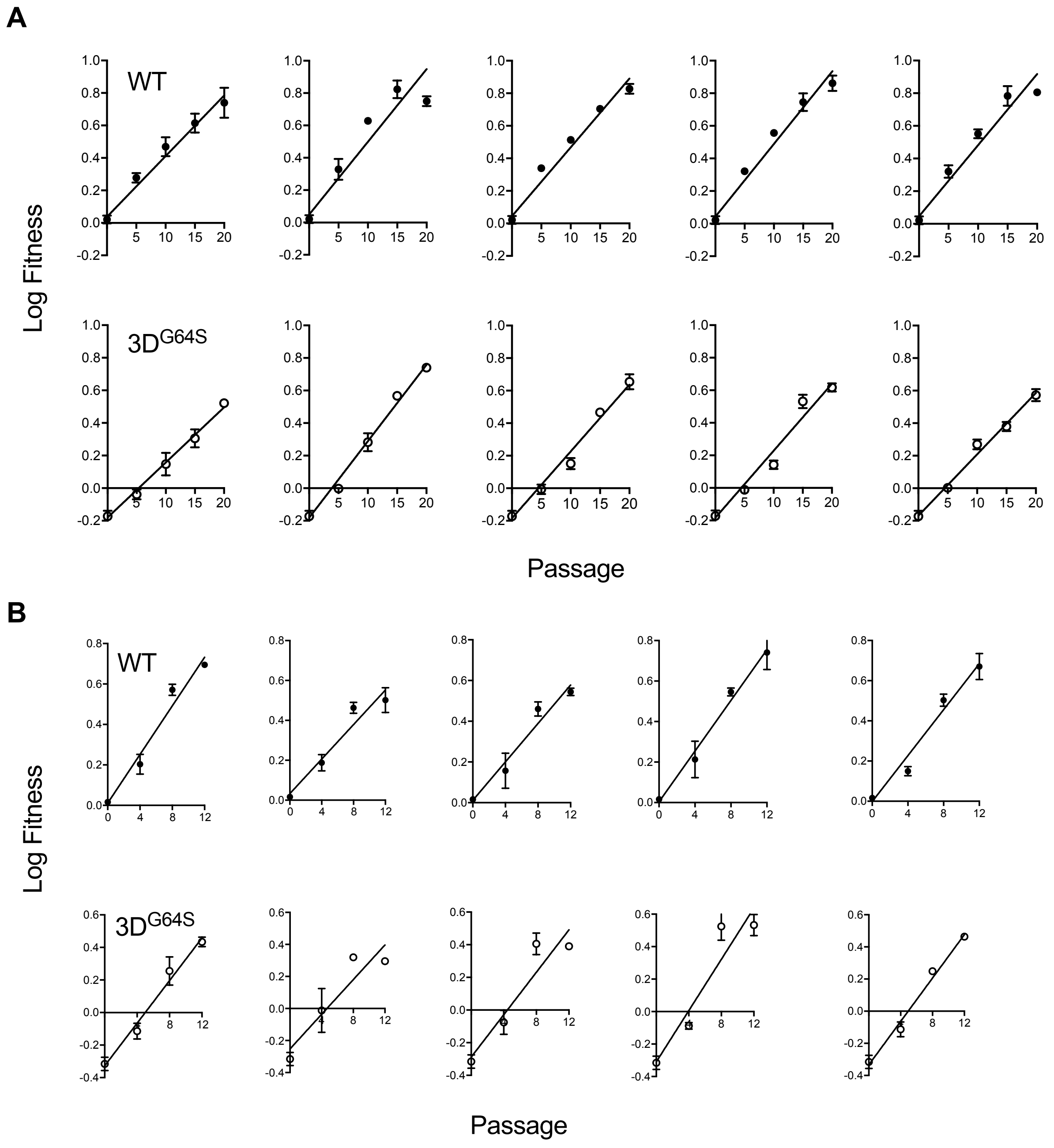

S2 Figure. Log10 fitness versus passage in adaptability experiment. Each point is the fitness mean ± standard deviation of three replicate competition assays. (A) WT and 3DG64S on HeLa (B) WT and 3DG64S on PVR-3T3. For the graphs in (B), the relationship of log fitness vs. passage was not linear past passage 16, and only passages 1-12 are shown. All plotted data can be found in SI, S1 Data.
