# Supplementary material for: A speed–fidelity trade-off determines the mutation rate and virulence of an RNA virus
Source: PLoS Biol. 2018 Jun 28;16(6):e2006459. doi: 10.1371/journal.pbio.2006459 (PMC6040757; doi:10.1371/journal.pbio.2006459)
Supplement: S3 Fig — (PDF) [file pbio.2006459.s005.pdf]

Figure S3

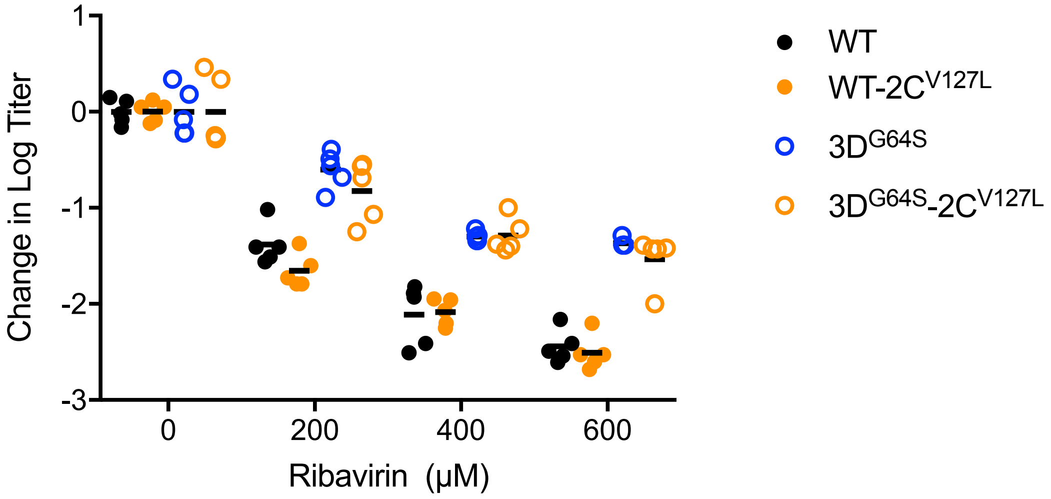

S3 Figure. Mutagen sensitivity of 2C-V127L variants. HeLa were infected at an MOI of 0.1 with the indicated viruses in presence of various concentrations of ribavirin. After 24 hours, titers of mock and ribavirin-treated populations were determined by TCID50 and those of ribavirin-treated populations were normalized to mock-treated controls (mean of 5 measurements per virus). Shown are the changes in titer (y-axis, mean, 5 replicates) for each virus at each drug concentration (x-axis). A greater reduction in titer (more negative number) indicates higher mutagen sensitivity and suggests a higher baseline mutation rate. The differences between G64S and G64S-V127L ($p=0.2204$) and WT and V127L at 200μM ribavirin ($p=0.0590$) were not significantly different (two sample t test with Welch correction). WT and G64S (which have a 2 fold difference in mutation rate by fluctuation test) did exhibit a statistically significant difference in mutagen sensitivity at 200μM ribavirin ($p=0.0003$). All plotted data can be found in SI, S1 Data.
