# Supplementary material for: A speed–fidelity trade-off determines the mutation rate and virulence of an RNA virus
Source: PLoS Biol. 2018 Jun 28;16(6):e2006459. doi: 10.1371/journal.pbio.2006459 (PMC6040757; doi:10.1371/journal.pbio.2006459)
Supplement: S1 Table — (DOCX) [file pbio.2006459.s006.docx]

**S1 Table.** Mutations conferring resistance to 1mM guanidine. Shown are results from 15 plaques from populations treated with no drug (WT) or 200µM ribavirin (R).

| Plaque | Mutations (position, nucleotide change, amino acid change) |  | Plaque 200µM R | Mutations (position, nucleotide change, amino acid change) |
| --- | --- | --- | --- | --- |
| WT2 | 4614 U->A, F->Y |  | R1 | 4676 G->U, A->S |
| WT6 | 4614 U->A, F->Y |  | R8 | 4676 G->U, A->S |
| WT12 | 4614 U->A, F->Y |  | R10 | 4676 G->U, A->S |
| WT14 | 4676 G->U, A->S |  | R12 | 4676 G->U, A->S |
| WT1 | 4682 A->U, M->L |  | R13 | 4676 G->U, A->S |
| WT3 | 4682 A->U, M->L |  | R7 | 4363 C->U, VAL(SYN) ; 4682 A->C, M->L |
| WT4 | 4682 A->U, M->L |  | R3 | 4702 G->A, M->I |
| WT5 | 4682 A->U, M->L |  | R2 | 4802 A->C, I->L |
| WT9 | 4682 A->U, M->L |  | R4 | 4802 A->C, I->L |
| WT10 | 4682 A->U, M->L |  | R5 | 4802 A->C, I->L ; 4810 C->U,PRO(SYN) |
| WT11 | 4682 A->U, M->L |  | R6 | 4797 G->A,SER(SYN) ; 4820 G->A, A->U |
| WT7 | 4702 G->A, M->I |  | R9 | 4820 G->U, A->S |
| WT13 | 4820 G->U, A->S |  | R11 | 4459 A->G, I->M ; 4820 G->U, A->S |
| WT8 | 4442 A->G,U->A ; 4823 C->A,H->N |  | R14 | 4820 G->U, A->S |
| WT15 | NONE in 2C |  | R15 | 4823 C->A, H->N |
