# Supplementary material for: A speed–fidelity trade-off determines the mutation rate and virulence of an RNA virus
Source: PLoS Biol. 2018 Jun 28;16(6):e2006459. doi: 10.1371/journal.pbio.2006459 (PMC6040757; doi:10.1371/journal.pbio.2006459)
Supplement: S2 Table — (DOCX) [file pbio.2006459.s007.docx]

**S2 Table.** Number, age, and sex of mice used in all experiments.

| Expt | Route | Dose | Age Range | WT | 3D^G64S^ | 3D^G64S^-2C^V127L^ | 3D^G64S ;I92T; K276R^ |
| --- | --- | --- | --- | --- | --- | --- | --- |
| IV1043 | IM | 10^6^ | 6w3d-8w6d | 18 (8♂ , 10♀) | 18 (9♂ , 9♀) | 18 (9♂ , 9♀) |  |
| IV1044 | IV | 10^7^ | 6w6d-8w4d | 15 (6♂ , 9♀) | 16 (6♂ , 10♀) | 16 (6♂ , 10♀) |  |
| IV1045 | IM | 10^5^ | 6w1d-8w3d | 12 (6♂ , 6♀) | 12 (6♂ , 6♀) | 12 (6♂ , 6♀) |  |
| IV1048 | IM | 10^4^ | 6w4d-7w1d | 12 (6♂ , 6♀) | 12 (6♂ , 6♀) |  |  |
| IV1049 | IM | 10^7^ | 7w-7w2d | 18 (11♂ , 7♀) | 18 (11♂ , 7♀) |  |  |
| IV1052 | IM | 10^6^ | 6w-7w6d | 16 (7♂ , 9♀) | 18 (8♂ , 10♀) |  |  |
| IV1053 | IV | 10^7^ | 6w3d-8w1d | 10 (4♂ , 6♀) | 10 (5♂ , 5♀) |  |  |
| IV1054 | IM | Var^a^ | 6w3d-8w1d | 24 (12♂ , 12♀) | 24 (13♂ , 11♀) |  |  |
| IV1055 | IM | 10^6^ | 7w-7w4d |  | 25 (11♂ , 14♀) | 25 (11♂ , 14♀) |  |
| IV1056 | IM | 10^6^ | 6w-6w3d |  | 25 (12♂ , 13♀) |  | 25 (13♂ , 12♀) |
| IV1057 | IM | 10^6^ | 6w3d-7w2d |  | 18 (9♂ , 9♀ ) |  | 18 (9♂ , 9♀ ) |

^a^ Varied dose for LD50 estimation. See Table S3
