# Supplementary material for: A speed–fidelity trade-off determines the mutation rate and virulence of an RNA virus
Source: PLoS Biol. 2018 Jun 28;16(6):e2006459. doi: 10.1371/journal.pbio.2006459 (PMC6040757; doi:10.1371/journal.pbio.2006459)
Supplement: S3 Table — (DOCX) [file pbio.2006459.s008.docx]

**S3 Table.** Raw data for calculation of LD50 (PD50, as paralysis triggered euthanasia per protocol). Calculation and output were obtained using tsk package in R with no trimming. Similar values were obtained using the logit method.

|  | 10^2^ pfu | 10^3^ pfu | 10^4^ pfu | 10^5^ pfu | 10^6^ pfu | 10^7^ pfu | PD50 | 95% CI | |
| --- | --- | --- | --- | --- | --- | --- | --- | --- | --- |
| WT | 0/6 | 0/6 | 3/6 | 5/6 | 6/6 | 6/6 | 1.47E+05 | 4.7E+04 | 4.6E+05 |
| 3D^G64S^ | ND | 0/6 | 1/6 | 3/6 | 6/6 | 6/6 | 6.81E+05 | 2.2E+05 | 2.1E+06 |
| Age range 6w3d-8w1d; 25♂ , 23♀ | | | |  |  |  |  |  |  |
| All animals dosed intramuscularly | | | |  |  |  |  |  |  |
| PD50 by Spearman-Karber method | | | |  |  |  |  |  |  |
